# Melatonin Modulates Endoplasmic Reticulum Stress and Akt/GSK3-Beta Signaling Pathway in a Rat Model of Renal Warm Ischemia Reperfusion

**DOI:** 10.1155/2015/635172

**Published:** 2015-07-01

**Authors:** Kaouther Hadj Ayed Tka, Asma Mahfoudh Boussaid, Mohamed Amine Zaouali, Rym Kammoun, Mohamed Bejaoui, Sonia Ghoul Mazgar, Joan Rosello Catafau, Hassen Ben Abdennebi

**Affiliations:** ^1^Unit of Molecular Biology and Anthropology Applied to Development and Health (UR12ES11), Faculty of Pharmacy, University of Monastir, rue Avicenne, 5000 Monastir, Tunisia; ^2^Laboratory of Histology and Embryology, Faculty of Dental Medicine, University of Monastir, rue Avicenne, 5000 Monastir, Tunisia; ^3^Unit of Experimental Hepatic Ischemia-Reperfusion, Institute of Biomedical Investigations, Higher Council of Scientific Investigations, 08036 Barcelona, Spain

## Abstract

Melatonin (Mel) is widely used to attenuate ischemia/reperfusion (I/R) injury in several organs. Nevertheless, the underlying mechanisms remain unclear. This study was conducted to explore the effect of Mel on endoplasmic reticulum (ER) stress, Akt and MAPK cascades after renal warm I/R. Eighteen Wistar rats were randomized into three groups: Sham, I/R, and Mel + I/R. The ischemia period was 60 min followed by 120 min of reperfusion. Mel (10 mg/kg) was administrated 30 min prior to ischemia. The creatinine clearance, MDA, LDH levels, and histopathological changes were evaluated. In addition, Western blot was performed to study ER stress and its downstream apoptosis as well as phosphorylation of Akt, GSK-3*β*, VDAC, ERK, and P38. Mel decreased cytolysis and lipid peroxidation and improved renal function and morphology compared to I/R group. Parallely, it significantly reduced the ER stress parameters including GRP 78, p-PERK, XBP 1, ATF 6, CHOP, and JNK. Simultaneously, p-Akt level was significantly enhanced and its target molecules GSK-3*β* and VDAC were inhibited. Furthermore, the ERK and P38 phosphorylation were evidently augmented after Mel administration in comparison to I/R group. In conclusion, Mel improves the recovery of renal function by decreasing ER stress and stimulating Akt pathway after renal I/R injury.

## 1. Introduction

Ischemia/reperfusion (I/R) injury remains a major problem encountered in vascular surgery, organ procurement, and transplantation and can lead to structural and functional cell damage [1]. In renal disease, I/R represents the most frequent cause of acute kidney injury [2]. The last years have witnessed a burgeoning development in our understanding of the molecular pathways involved in I/R and various mechanisms have been proposed to explain the origins of tissue injury. It is generally believed that reactive oxygen species (ROS) are key mediators of the I/R induced damage to the kidney. The excessive formation of ROS activates a host of signaling pathways including endoplasmic reticulum (ER) stress and cell death [3].

ER plays an important role in synthesis and maturation of proteins, biosynthesis of lipids, regulation of calcium, and maintenance of cell homeostasis [4]. Disturbances such as hypoxia, glucose starvation, and oxidative stress may lead to ER disorder which can provoke ER stress. Subsequently, a signal transduction cascade termed the unfolded protein response (UPR) is induced [3]. UPR is an adaptive response that aims to restore normal ER function. It comprises three branches: activating transcription factor (ATF) 6, inositol-requiring enzyme (IRE) 1, and RNA activated protein kinase- (PKR-) like ER kinase (PERK). These proteins are normally held in inactive states in ER membranes by binding to an intra-ER chaperone, the glucose regulated protein (GRP) 78. In response to stress, GRP78 dissociates from ER membrane to bind misfolding proteins freeing in this way, ATF6, IRE1, and PERK, which initiate signal transduction processes in order to reestablish ER homeostasis [5]. Though UPR normally helps in cell survival by removing misfolded proteins, an elevated and prolonged ER stress level can cause cell death [4]. Consequently, this can contribute to a diverse range of pathophysiological events including acute and chronic renal disease [3]. Hence, therapeutic strategies targeting ER stress and its downstream apoptosis might have the potential to provide a powerful tool in an attempt to reduce I/R injury.

Melatonin (N-acetyl-5-methoxytryptamine) (Mel) is a hormone secreted by pineal gland and is mainly responsible for controlling circadian cycle [6]. It is a highly lipophilic molecule that crosses cell membranes to easily reach subcellular compartments [7]. This small amphiphilic molecule and its metabolites are likewise potent scavengers of ROS [8]. Apart from this, Mel was shown to possess anti-inflammatory and antiapoptotic actions [9, 10] as well as other remarkable cell protective properties [11, 12]. The efficacy of Mel treatment to reduce renal I/R injury has been well-documented. However, experiments evaluating the underlying mechanisms of action, particularly its effect on ER stress and apoptosis, are lacking. Therefore, the present study was made to investigate whether Mel can influence the I/R induced ER stress in the kidney and whether its renoprotective effect implicates the activation of prosurival signaling cascades including Akt/GSK-3*β* pathway.

## 2. Materials and Methods

### 2.1. Animals and Experimental Groups

Male Wistar rats weighing 200–250 g were used in this study. Animals were maintained at constant temperature (23 ± 2°C) with a 12 h light-dark cycle and free access to water and food. All procedures were carried out in accordance with the European Union Regulations (Directive 86/609/CEE) for animal experiments. Animals were randomly assigned into the following experimental groups, each containing 6 rats.

*I/R Group.* Rats had been injected with a vehicle solution consisting of ethanol and NaCl 0.9% mixture (the final concentration of ethanol was 1%) intraperitoneally (i.p.) 30 min before they were subjected to bilateral renal ischemia. The renal pedicles were occluded for 60 min using nontraumatic vascular clips, followed by reperfusion for 120 min [13]. 

*Mel + I/R Group*. This group was the same as I/R group but animals were treated with Mel (10 mg/kg i.p.) 30 min before renal clamping [6, 14]. Mel (Sigma Chemical, St. Louis, MO, USA) was dissolved in the vehicle solution. 

*Sham Group*. Animals were subjected to the same surgical procedure described above but were not subjected to renal I/R and did not receive any treatment.

### 2.2. Surgery and Experimental Protocols

As described previously by Mahfoudh-Boussaid et al. [13] rats were anesthetized through an intraperitoneal injection of ketamine hydrochloride (50 mg/kg) and xylazine (10 mg/kg) and placed onto a thermostatically controlled warm board to maintain body temperature at 37°C. After performing a midline laparotomy, the renal pedicles containing the renal artery, vein, and nerves supplying each kidney were carefully isolated and occluded. After clamps' removal, the bladder was cannulated for the collection of urine during the last 30 min of reperfusion and the abdomen was closed in layers. The mean arterial pressure was measured using a pressure transducer (Pression Monitor BP-1; Pression Instruments, Sarasota, FL) connected to the right carotid artery. The left jugular vein was cannulated for mannitol (10%) and heparin (50 U/mL) infusion (Minipuls 3 peristaltic pump, Gilson, France).

At the end of the reperfusion period, rats were euthanized and blood samples were collected via carotid artery. Simultaneously, both kidneys were harvested and weighed. Plasma, urine, and tissue samples were immediately kept at −80°C for biochemical and Western blot analyses.

### 2.3. Assessment of Renal Function

The renal function was evaluated during the last thirty minutes of reperfusion by calculating the creatinine clearance using the standard formula:

(1) Creatinine  clearance  μL/min/g  of  weight=Creat  U∗VCreat  P.

Creat U is the creatinine concentration in urine (*μ*mol/L), V is urine flow rate (*μ*L/min/g of weight), and Creat P is the creatinine concentration in plasma (*μ*mol/L).

Serum and urine creatinine concentrations were measured spectrophotometrically at 490 nm by the Jaffé kinetic reaction (BioMerieux Kit, France).

### 2.4. LDH Assay

The activity of lactate dehydrogenase (LDH) in the plasma was quantified using LDH assay kit (Spinreact, Spain).

### 2.5. Lipid Peroxidation Assessment

Malondialdehyde (MDA) is an end product of peroxidation of cell membrane lipids caused by oxygen derived free radicals. It was measured in renal tissue using the thiobarbiturate reaction at 530 nm [15].

### 2.6. Renal Histology

Samples from all kidneys were fixed in 10% formalin solution and processed for histology by hematoxylin and eosin (H&E) staining. Histological evaluations were performed using light microscopy at a magnification of 40–400 by an experienced renal pathologist without having knowledge about the treatment groups. Morphological changes from the whole cross-sectional area of the kidneys were assessed and a semiquantitative analysis of histological damage was conducted. A score from 0 to 4 was given to assess necrosis according to the method of Jablonski et al. [16] as the following: 0, normal; 1, necrosis of individual cells; 2, necrosis of all cells in adjacent proximal convoluted tubule (PCT), with survival of surrounding tubules; 3, necrosis confined to distal third PCT with bands of necrosis extending across inner cortex; 4, necrosis of all three segments of PCT.

### 2.7. Western Blot Analysis

Western blot analysis was performed as previously described by Mahfoudh-Boussaid et al. [13]. Briefly, tissue samples from the kidneys were prepared with lysis buffer (150 mM NaCl, 50 mM Tris HCl, 1 mM DTT, 50 mM NaF, 1 mM PMSF, 1 mM EDTA, 1 mM EGTA, 0.1 mM orthovanadate, 0.05% Triton X-100, and 2% protease inhibitor cocktails). Protein concentrations were determined according to the Bradford method. Protein extracts (50 *μ*g/lane) were then separated by SDS-PAGE electrophoresis and transferred to polyvinylidene fluoride membranes. Membranes were immunoblotted with antibodies directed against GRP78, total and p-PERK, XBP-1, ATF6*α* and CHOP (Santa Cruz Biotechnology, Santa Cruz, CA, USA), *β*-actin (Sigma chemical, St. Louis, MO), total and p-JNK, total and p-Akt, total and p-GSK-3*β*, total and p-ERK, total and p-P38 (Cell Signalling Technology Inc., Beverly, MA, USA), and total and p-VDAC. The bands were visualized using an enhanced chemiluminescence kit (Bio Rad Laboratories, Hercules, CA, USA). The values were obtained by densitometric scanning and the Quantity One software program (Bio Rad Laboratories, Hercules, CA, USA).

### 2.8. Statistical Analysis

The data are presented as means ± SEM. Statistical analysis was performed using one-way ANOVA, followed by Newman-Keuls multiple comparisons. *p* < 0.05 was considered statistically significant.

## 3. Results

**Figure 1:**
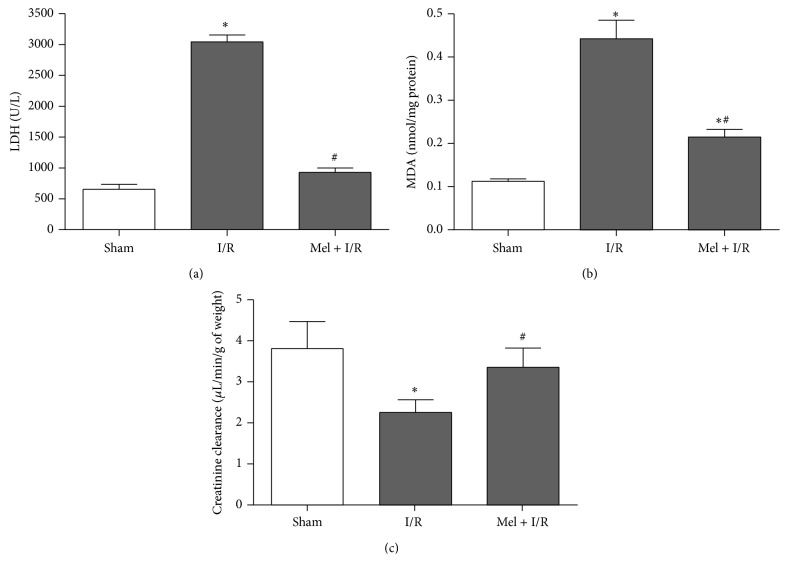
Evaluation of lactate dehydrogenase activity in plasma (a), malondialdehyde concentration in tissue (b), and creatinine clearance (c). Results are expressed as mean ± SEM (*n* = 6 for each group).  ^*∗*^ *p* < 0.05 versus Sham. ^#^ *p* < 0.05 versus I/R.

Cytolysis was assessed by measuring the activity of LDH in the plasma (Figure 1(a)). Level of LDH increased from baseline values of 654 ± 81 U/L in Sham to 3044 ± 109 U/L in the I/R group (*p* < 0.05). This rise was significantly lower after Mel treatment with values of 930 ± 69 U/L compared to I/R group (*p* < 0.05).

Then, we examined the protective effect of Mel on lipid peroxidation (Figure 1(b)). Renal I/R significantly increased MDA level to 0.44 ± 0.04 nmol/mg protein compared with nontreated group where MDA concentration was 0.11 ± 0.01 nmol/mg protein (*p* < 0.05). After the administration of Mel, we observed a significant drop in this parameter reaching 0.21 ± 0.02 nmol/mg protein regarding I/R group (*p* < 0.05).

Renal function was evaluated comparing the variation of creatinine clearance among the three groups (Figure 1(c)). Kidneys from I/R group revealed a dramatic decrease in creatinine clearance with values of 2.3 ± 0.2 *μ*L/min/g of weight compared to those of Sham group with values of 3.8 ± 0.4 *μ*L/min/g of weight (*p* < 0.05). An important functional recovery was observed subsequently to Mel administration prior to renal I/R and the creatinine clearance was 3.36 ± 0.3 *μ*L/min/g of weight (*p* < 0.05) in comparison to I/R group.

**Figure 2:**
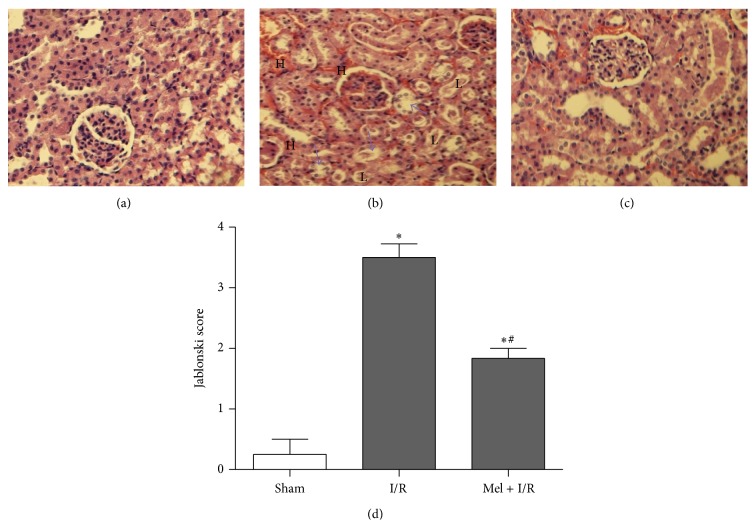
Representative histological photographs of kidney tissues from Sham (a), I/R (b), and Mel + I/R (c) groups (H&E) ×400. Semiquantitative assessment of renal necrosis among the different experimental groups using the Jablonski score (d). The arrows denote brush border loss, “H” denotes hemorrhage, and “L” denotes nuclei loss. Results are expressed as mean ± SEM (*n* = 6 for each group).   ^*∗*^ *p* < 0.05 versus Sham.  ^#^ *p* < 0.05 versus I/R.

Histological changes were in keeping with biochemical parameters of renal injury. The morphological examination of all groups confirmed that there was renal impairment by severe tubular damage after I/R (Figure 2(b)) compared to Sham group (Figure 2(a)). These features included brush border loss, nuclear condensation, cell swelling, a consistent loss of nuclei, and hemorrhage. Renal sections obtained from rats pretreated with Mel demonstrated a marked reduction of the histological features of renal injury (Figure 2(c)). The Jablonski score (Figure 2(d)) in the I/R rat kidney was 3.5 ± 0.2 versus 0.33 ± 0.33 in the Sham kidney (*p* < 0.05). In comparison to the I/R group, treatment with Mel significantly attenuated this score reaching 1.83 ± 0.2 (*p* < 0.05).

**Figure 3:**
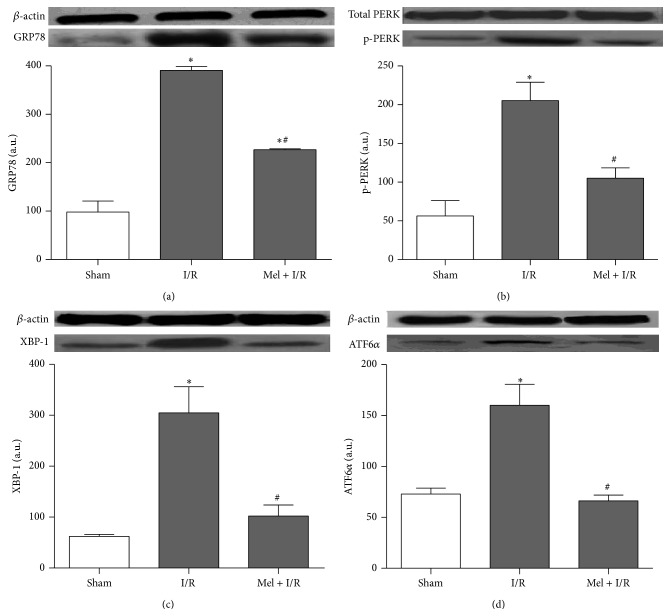
Western blot of GRP 78 (a), total and phosphorylated PERK (b), XBP-1 (c), and ATF6*α* (d). *β*-actin was used as a loading control. One representative blot of six independent experiments is shown at the top whereas densitometric analysis is shown at the bottom. Results are expressed as mean ± SEM.   ^*∗*^ *p* < 0.05 versus Sham.   ^#^ *p* < 0.05 versus I/R.

Afterwards, we examined the possible involvement of Mel in modulating ER stress induced by I/R injury. Different ER stress pathways were explored (Figure 3). Renal I/R significantly increased the relative amounts of GRP78, p-PERK, XBP-1, and ATF6*α* from Sham values of 98 ± 23, 56 ± 20, 62 ± 4, and 73 ± 61, respectively, to 391 ± 8, 205 ± 24, 305 ± 51, and 160 ± 21, respectively (*p* < 0.05). Interestingly, rats undergoing Mel treatment demonstrated a lower level activation of these ER stress parameters in the kidney compared with their respective I/R groups with values of 227 ± 2, 105 ± 13, 102 ± 22, and 66 ± 5, respectively (*p* < 0.05).

**Figure 4:**
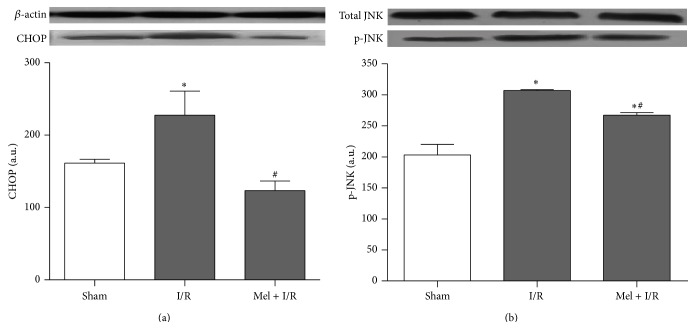
Western blot of CHOP (a) and total and phosphorylated JNK (b). The *β*-actin was used as a loading control. One representative blot of six independent experiments is shown at the top whereas densitometric analysis is shown at the bottom. Results are expressed as mean ± SEM.   ^*∗*^ *p* < 0.05 versus Sham.   ^#^ *p* < 0.05 versus I/R.

In line with this, we studied the ER stress induced apoptosis by focusing on two proapoptotic parameters. As indicated in Figure 4, renal I/R markedly enhanced the activation of CHOP as well as JNK and the respective values were 228 ± 33 and 307 ± 1 versus 161 ± 5 and 203 ± 17 for Sham (*p* < 0.05). In contrast, this effect was significantly attenuated by preischemic Mel administration and values dropped to 123 ± 13 and 267 ± 4, respectively, in comparison to I/R solely.

**Figure 5:**
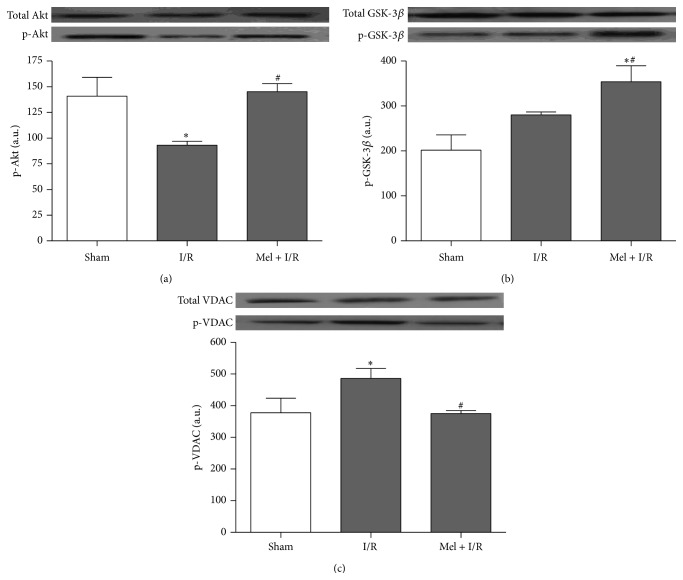
Western blot of total and phosphorylated Akt (a), total and phosphorylated GSK-3*β* (b), and total and phosphorylated VDAC (c). One representative blot of six independent experiments is shown at the top whereas densitometric analysis is shown at the bottom. Results are expressed as mean ± SEM.  ^*∗*^ *p* < 0.05 versus Sham.  ^#^ *p* < 0.05 versus I/R.

Furthermore, we evaluated the influence of Mel on some targets of the Akt signaling pathway. We found that Akt phosphorylation was notably decreased after I/R to values of 93 ± 4 when compared to Sham where p-Akt level was 141 ± 18 (*p* < 0.05) (Figure 5(a)). This was concomitant with a significant increase in phosphorylated VDAC amount with values of 486 ± 31 as compared to Sham with values of 366 ± 35 (*p* < 0.05) (Figure 5(c)). However, there was no obvious difference between I/R and sham groups regarding GSK-3*β* phosphorylation (Figure 5(b)). The administration of Mel was found to reverse the effect of I/R on Akt and VDAC phosphorylation and thus p-Akt level rose noticeably reaching 145 ± 8 whereas p-VDAC amount dropped until 375 ± 9 (*p* < 0.05). Moreover, GSK-3*β* phosphorylation was significantly enhanced after Mel treatment to 354 ± 36 in comparison to both other groups.

**Figure 6:**
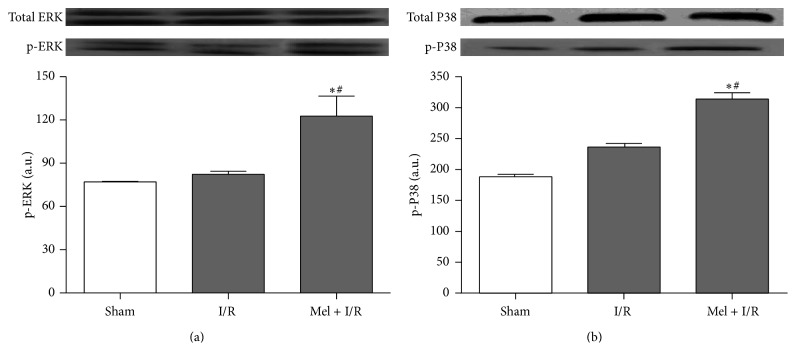
Western blot of total and phosphorylated ERK (a) and total and phosphorylated P38 (b). One representative blot of six independent experiments is shown at the top whereas densitometric analysis is shown at the bottom. Results are expressed as mean ± SEM.  ^*∗*^ *p* < 0.05 versus Sham.  ^#^ *p* < 0.05 versus I/R.

To finally explore the relevance of Mel treatment on some MAPK features after exposure to renal I/R, immunoblot analyses of ERK and P38 in kidneys from all groups were performed. As shown in Figure 6, our results reveal no significant differences between Sham and I/R groups regarding phosphorylated ERK and P38 levels. However, Mel treatment resulted in a significant enhancement in these two parameters with values of 123 ± 14 and 314 ± 18, respectively (*p* < 0.05), in comparison to both other groups.

The effect of Mel without I/R (Sham + Mel group) was assessed regarding the parameters used in the present study (data not shown) and no significant differences were recorded in comparison with Sham group. Therefore, only the data obtained in Sham group were mentioned in this study and were used for further statistical analysis with I/R and Mel + I/R groups.

## 4. Discussion

Renal I/R injury remains an unresolved problem that has immediate and deleterious effects in both native and transplanted kidneys [17]. The pathogenesis underlying I/R injury is complex involving different molecular pathways which are not fully understood [18]. The present study investigated the effects of Mel on renal I/R injury. We showed that preischemic treatment with Mel significantly reduced I/R induced injury in the rat kidney. The current data are in agreement with previous results demonstrating that Mel pretreatment protects against warm I/R injury in a variety of tissues and organs, including the heart [19], liver [20], brain [21], and kidney [22, 23].

In addition to the decrease of I/R induced elevation in lipid peroxidation, our findings revealed that Mel reduced LDH release and improved the creatinine clearance of ischemic kidneys. It has been suggested that lipid peroxidation is closely related to I/R induced tissue damage and that MDA is an indicator of lipid peroxidation rate [24]. The peroxidation of membrane lipids can disrupt membrane fluidity and cell compartmentalization which may result in cell lysis [25] and thus LDH release. It is well-recognized that Mel has antioxidant effects and its role in lipid peroxidation inhibition has been well-established [26, 27]. Besides, the effect of Mel on LDH leakage suggests that it appears to preserve the integrity of cell membranes and renal architecture as well. This may, in part, explain the important function recovery observed after Mel administration.

The next finding of this study shows that Mel significantly attenuated the level of several ER stress parameters induced by renal I/R. It is well-documented that ER stress plays a significant role in the pathogenesis of renal I/R injury [28, 29]. In line with this, Mahfoudh-Boussaid et al. [13] have described that renal I/R is concomitant with amplified levels of GRP 78, p-PERK, ATF4, and XBP-1. These data are in agreement with the present investigation showing that renal I/R induced elevations in GRP78, XBP-1, p-PERK, CHOP, and ATF6. Interestingly, Mel treatment was found to counteract these elevations. Our results are in keeping with recent findings demonstrating that melatonin reduces ER stress in different models of cell injury. For instance, Mel represses the UPR in rabbits with lethal fulminant hepatitis [30]. Similar effects were also observed in the steatotic liver [31]. Furthermore, a marked reduction of ER stress after Mel treatment was also observed in brain of rats subjected to arsenite-induced neurotoxic conditions [32]. However, whether Mel is involved in protection against ER stress in warm renal I/R injury is unclear. In this report, we demonstrate for the first time the reduction of ER stress after melatonin treatment in this experimental model of renal I/R. The effectiveness of Mel in reducing ER stress was also associated with attenuation of cell death as evidenced by lower levels of CHOP and p-JNK. CHOP and JNK activation has been correlated with apoptosis as an ER stress downstream event [33, 34]. Increasing evidence has shown that induction of CHOP is an important element of switch from prosurvival to proapoptotic signaling cascades [35, 36]. In this same context, activation of JNK was correlated with triggering apoptotic signaling mechanisms following I/R injury in various organs [37, 38]. In light of the findings that Mel reduced ER stress induced apoptosis, we hypothesized that this cell death attenuation would be in favor of survival enhancement.

To further elucidate the mechanisms by which Mel modulates cell survival during renal I/R, we evaluated its effects on Akt and its downstream targets, GSK-3*β* and VDAC. Results of the current study showed that p-Akt decreased in the case of I/R injury and that Mel prevented this downregulation. Many researchers have demonstrated that Akt plays a crucial role in the protection of liver [39], heart [40] and kidney [41] against I/R injury. One of the proposed mechanisms is that Akt phosphorylates GSK-3*β* which decreases the level of phosphorylated VDAC, the most abundant protein in mitochondrial outer membrane, leading to inhibition of the apoptotic process [13, 42]. The capability of Mel to enhance Akt activation in the setting of I/R has been documented in several organs including brain [43] and liver [44] but is not yet proven in kidney. Our data suggest that antiapoptotic effect of Mel is mediated, in part, by Akt signaling axis activation during renal I/R.

There are likely many other mechanisms by which Mel can promote cell survival. In order to explore other possible pathways, we examined the phosphorylation levels of ERK and P38 which are serine/threonine kinases belonging to the MAPK family. ERK activation is commonly considered as protective in the setting of renal I/R [45]. Besides, some studies report that p-ERK could be responsible, at least partially, for the inhibition of GSK-3*β* activity [46] thus contributing to apoptosis decrease. Our results indicated that preconditioning with Mel induced a significant increase in p-ERK level regarding kidneys undergoing I/R injury. This is in keeping with a previous report demonstrating that Mel effectively promotes ERK phosphorylation in case of cerebral I/R [47]. P38 is a crucial signaling protein that is involved in cellular proliferation, differentiation, inflammation, and apoptosis [48]. According to our findings, Mel administration results in a significant activation of P38. While the evidence is mounting that P38 inhibition might be beneficial in reducing inflammation and I/R injury [49]. Other reports suggest that P38 activation may confer protection [50]. Importantly, some of the downstream targets of P38 are protective, while others are inducers of cell death, and the overall result of P38 activation may depend on the balance between them [51]. Hernendez and coworkers identified a beneficial role for P38 in the setting of cardiac I/R [52]. It is obviously critical to be certain that P38 inhibition will not exacerbate I/R injury. Unfortunately, there is no clear answer to this question. Nevertheless, given that Mel is shown to reduce molecular damage and cellular loss, our hypothesis would be in favor of beneficial role of P38 signaling cascade regarding our renal I/R model. However, how Mel activates the protective aspects of P38 pathway will be a major issue to be addressed in the future.

## 5. Conclusion

On the basis of the previous findings, we conclude that Mel could ameliorate renal damage related to I/R. The underlying mechanisms of this protection essentially involve the modulation of ER stress, Akt and MAPK pathways.
